# Biomimetic Strategies in Orthosis Design: A Scoping Review of Biological Abstraction and Functional Translation

**DOI:** 10.3390/biomimetics11040241

**Published:** 2026-04-03

**Authors:** Tim Tchervonenko, Alexander Sauer, Thabata Alcântara Ferreira Ganga, Heike Beismann, Eduardo Keller Rorato, Míriam Raquel Diniz Zanetti, Maria Elizete Kunkel

**Affiliations:** 1 Institute for Biomimetics, Westphalian University of Applied Sciences, 46397 Bocholt, Germany; tim.tchervonenko@studmail.w-hs.de (T.T.); heike.beismann@w-hs.de (H.B.); 2 3D Orthotics and Prosthetics Laboratory, Science and Technology Institute, Federal University of São Paulo (UNIFESP), São José dos Campos 12247014, Brazil; thabata.ganga@unifesp.br (T.A.F.G.); ek.rorato@unifesp.br (E.K.R.); elizete.kunkel@unifesp.br (M.E.K.)

**Keywords:** biomimetic design, orthosis development, biological abstraction

## Abstract

Orthoses are widely used to support or modulate neuromuscular and skeletal function; however, their clinical effectiveness is often limited by discomfort, poor adaptability, and suboptimal human–device interaction. Biomimetics has emerged as a structured design paradigm capable of enhancing orthotic performance by systematically translating biological principles into engineering solutions. This scoping review examined biomimetic strategies in the development of orthoses. A structured search was conducted across PubMed, IEEE Xplore, Web of Science, and Scopus (2000–2025). Of 453 identified records, 14 met the inclusion criteria. Biomimetic orthosis research emerged predominantly after 2012, with increased activity after 2021. Human-based biological models, particularly muscle–tendon systems, predominated. Most studies relied on functional abstraction and were implemented using cable-driven or electromechanical actuation. None of the included studies explicitly referenced established biomimetics standards (e.g., ISO 18458), and descriptions of biological analysis, abstraction, and transfer were frequently incomplete. Experimental validation was generally limited to prototype-level testing, small sample sizes, and short-term evaluations, with no longitudinal or multicenter studies identified. These findings reveal a structural imbalance between conceptual biomimetic inspiration and structured methodological implementation. Based on this analysis, a structured biomimetic workflow is proposed to enhance traceability, reporting clarity, and clinical translation in the development of orthosis.

## 1. Introduction

Orthoses constitute a fundamental component of conservative and rehabilitative care, providing external devices designed to modify structural alignment, redistribute loads, or assist neuromuscular function [1]. Their clinical objectives include pain relief, mechanical stabilization, prevention or correction of deformities, and improvement of functional performance [2]. The relevance of orthotic devices extends beyond specialized clinical environments, as assistive products are required by a growing global population driven by demographic aging and the increasing prevalence of chronic and disabling conditions [3]. This expansion underscores the need for devices that are not only biomechanically effective but also comfortable, adaptable, and acceptable for long-term daily use. 

Despite their widespread prescription, the real-world effectiveness of orthoses is frequently constrained by limitations at the human–-device interface [4]. Discomfort, restricted mobility, aesthetic concerns, and limited integration into daily activities have been consistently reported across orthoses and related assistive technologies [5,6,7,8]. Clinical benefit, therefore, depends not only on mechanical performance but also on the integration of biomechanical intent with usability, ergonomics, and user adherence. These challenges underscore the need for innovation strategies that reconcile mechanical robustness with biological compatibility [9,10]. 

In this context, biomimetics has emerged as a structured, interdisciplinary design approach for improving engineered systems by translating biological principles into technical solutions [11]. Rather than relying on simple analogies, biomimetics focuses on identifying biological functions, abstracting the underlying mechanisms, and transferring them into engineering design [12,13,14,15,16,17]. International standardization efforts have sought to formalize biomimetic terminology and methodological workflows, particularly through the following ISO standards. ISO 18458 [11] defines the core concepts and terminology of biomimetics, including the processes of biological analysis, abstraction, and transfer into engineering design. Complementarily, ISO 18457 [12] provides guidance for the identification and analysis of biological models relevant to technical challenges in the field of materials, structures and components, while ISO 18459 [13] addresses structural optimization. Together, these standards aim to establish a structured methodological framework for the systematic transfer of biological principles into engineering solutions. However, the extent to which such structured biomimetic approaches have been adopted in orthosis development remains unclear.

In rehabilitation engineering, biomimetic concepts have informed the design of prosthetic limbs, wearable robotics, and bioinspired sensing systems [20,21,22,23,24,25]. Applications include muscle-like actuation, tendon-driven transmission systems, graded stiffness architectures, and load-adaptive structural configurations. When combined with computational modeling, topology optimization, and additive manufacturing, biomimetic strategies offer the potential for lightweight, multifunctional, and patient-specific orthotic constructions [17,20,21]. These approaches enable multifunctional and potentially patient-specific constructions [15]. These strategies have been proposed not only to enhance mechanical efficiency but also to improve the human–device interface by promoting comfort, usability, and adherence, thereby directly addressing key limitations associated with conventional rigid and standardized orthotic systems [26,27,28,29,30,31].

Despite this potential, the application of biomimetics to orthosis development remains fragmented. Although previous reviews have examined bioinspired approaches in prosthetics [20,32,33] and healthcare robotics [21,34], no focused synthesis has critically analyzed how biological models are selected, abstracted, and translated into orthotic design solutions. As a result, it remains unclear to what extent biomimetics has been applied in a systematic, transparent, and reproducible way across orthotic research.

This terminology-based strategy may also have excluded orthotic systems that incorporate biologically inspired principles without explicitly describing the design process as biomimetic or bioinspired. Accordingly, the present review should be interpreted as a mapping of term-explicit biomimetic orthosis research rather than an exhaustive representation of all orthotic systems that may implicitly draw on biological analogies.

To address this gap, the present scoping review aims to identify and critically synthesize studies that apply biomimetic principles to the development of orthoses. The review introduces a structured four-layer analytical framework, encompassing biological abstraction, engineering implementation, experimental validation, and technological maturity, to examine how biological inspiration is translated into orthotic systems and how far these systems progress toward clinical integration. By mapping research trends, identifying methodological inconsistencies, and assessing validation maturity, this review seeks to clarify the current state of biomimetic orthosis research and to inform future efforts toward more standardized, transparent, and clinically robust design methodologies [35].

## 2. Methods

### 2.1. Protocol and Reporting Framework

This scoping literature review followed a protocol-oriented approach based on the guidelines proposed by Kitchenham and Charters [36]. Reporting was conducted in partial accordance with the Preferred Reporting Items for Systematic Reviews and Meta-Analyses (PRISMA 2020) checklist [37].

The review protocol predefined the research question, eligibility criteria, information sources, search strategy, screening procedure, data extraction plan, and the biomimetics-focused methodological transparency assessment applied to the studies. Although the protocol was not registered in a public database (e.g., PROSPERO), all eligibility criteria, search strategies, and analytical procedures were predefined prior to study screening to reduce selection bias and enhance reproducibility. Registration was not pursued because the review does not evaluate clinical intervention outcomes but focuses on engineering design strategies and biomimetic abstraction processes, which fall outside the typical scope of registries dedicated to clinical effectiveness reviews. The present study, therefore, follows PRISMA guidance for scoping reviews within engineering and design research contexts. To structure the research scope, the PICOC framework (Population, Intervention, Comparison, Outcome, Context) was applied [38].

- Population: Orthotic devices and orthosis-related technologies developed using biomimetic principles;
- Intervention: Application of biomimetic principles to orthosis design;
- Comparison: Not formally defined;
- Outcome: Identification of research trends, abstraction strategies, engineering implementations, validation characteristics, and methodological gaps;
- Context: Biomedical and rehabilitation engineering research involving orthotic devices for therapeutic or assistive purposes.

Based on this framework, the guiding research question was formulated as: which publications examine the use of biomimetics in the development of orthoses, and what translational patterns and methodological gaps can be identified within this body of literature?

### 2.2. Identification of Studies

A structured search strategy was implemented across multiple indexing platforms to ensure broad coverage of the biomedical, rehabilitation, and engineering domains. The following databases were searched: PubMed, IEEE Xplore, Web of Science, and Scopus. All searches were conducted on 25 September 2025. In addition to database searching, backward reference screening of the included studies was performed to identify potentially relevant publications. The search period covered publications published from 2000 to 2025. This temporal restriction was adopted to capture research emerging after the consolidation of biomimetics as a formalized interdisciplinary methodology, as well as during the rapid expansion of additive manufacturing, soft robotics, and computational modeling technologies relevant to orthotic systems. All retrieved records were imported into Zotero 6 for duplicate detection and reference management.

### 2.3. Search Strategy and Eligibility Criteria 

The search strategy combined orthosis-related terms with biomimetics-related descriptors using Boolean operators. The base query was defined as follows: (“orthosis” OR “orthoses” OR “orthotic” OR “brace” OR “splint”) AND (“biomimetic” OR “biomimicry” OR “bio-inspired” OR “biologically inspired” OR “bionic”). Database-specific adaptations were applied to account for differences in indexing structures and search syntax.

Studies were included if they met all of the following criteria:

- Published between 2000 and 2025;
- Full text available in English, German, Portuguese, or Spanish;
- Explicit use of biomimetic terminology (biomimetic, biomimicry, bio-inspired, biologically inspired, or bionic) directly associated with orthosis-related terminology;
- Classified as original research, technical investigations, clinical pilot studies, or systematic reviews;
- Contributed fully or partially to addressing the guiding research question.

Studies were excluded if they met any of the following criteria:

- Exclusive focus on prostheses without relevance to orthotic devices;
- Publications of three pages or fewer, as short-format contributions often lack sufficient methodological detail regarding biological abstraction, engineering translation, and validation processes;
- Editorials, commentaries, or opinion pieces;
- Full text unavailable.

The explicit requirement for biomimetic terminology was adopted to ensure conceptual clarity; however, this criterion may have excluded studies employing biomimetic principles without explicitly labeling them as such.

### 2.4. Study Selection Process and Reliability Procedures 

The selection of studies followed a multistage process: duplicate removal, title and abstract screening, and full-text eligibility assessment. Titles and abstracts were screened to identify potentially relevant studies, followed by full-text assessment of the remaining articles to determine final eligibility. The initial screening was conducted by a single reviewer. In cases of uncertainty regarding eligibility, decisions were discussed with the co-authors to ensure the consistent application of the inclusion criteria. To structure screening and ensure traceability, records were organized into three analytical categories:

(i) Biomimetic orthoses—Orthotic devices explicitly developed using biological inspiration and abstraction;
(ii) Biomimetic materials, tools, or mechanisms relevant to orthosis development—Studies presenting biologically inspired components applicable to orthotic systems;
(iii) Non-eligible studies—Studies neither orthotic nor biomimetic.

Titles and abstracts were used to assign each record to one of the three categories. Full-text assessment was subsequently conducted for studies classified under categories (i) and (ii). To mitigate potential selection bias, a test–retest reliability procedure was implemented. A random sample of 100 records was re-evaluated at the title and abstract stage after a 7-day interval to assess decision consistency. Additionally, 20 studies from category (i) and 30 from category (ii) were re-reviewed at the full-text stage after a 7-day interval. In cases of discrepancy, up to three additional re-evaluation rounds were conducted (2-day intervals between rounds). Nevertheless, the absence of independent dual screening represents a methodological limitation and should be considered when interpreting the findings.

### 2.5. Data Extraction and Methodological Transparency Assessment 

Data extraction was conducted using a predefined extraction form. This form was developed to systematically capture variables relevant to the identification of translational patterns and methodological gaps (Supplementary Table S1, Data Extraction Template). It was piloted on seven included studies and refined prior to full data extraction. The extracted variables included:

- Bibliographic information (title, authors, year);
- Biological reference model;
- Level of abstraction (morphological, functional, control-based, material-inspired);
- Anatomical region;
- Orthosis classification;
- Actuation category and energy source;
- Structural architecture;
- Personalization strategy;
- Validation characteristics (testing level, participants, follow-up);
- Reported objectives, outcomes, and limitations.

Missing information was recorded as “not reported.” Study authors were not contacted to obtain additional data. As the objective of this review was to map translational patterns rather than estimate clinical effect sizes, a conventional risk-of-bias assessment was not performed. Given that the included studies ranged from conceptual design papers and simulation-based investigations to feasibility-oriented prototype testing, the use of a conventional clinical risk-of-bias tool was considered not methodologically appropriate. Instead, the review employed a targeted assessment of biomimetic methodological transparency and translational reporting maturity, focusing on the extent to which biological analysis, abstraction, technical transfer, and validation characteristics were explicitly documented. This assessment was guided by two core questions:

(1) Was an explicit reference to a biomimetics standard (e.g., ISO 18458) provided?
(2) Were biological analysis, abstraction, and transfer processes explicitly described?

This assessment was designed to evaluate the maturity of the reports, not to quantify the quality of the studies.

### 2.6. Synthesis Strategy 

The synthesis examined categorical distributions across extracted variables to identify patterns in biological abstraction, engineering implementation, validation maturity, and reporting transparency. Quantitative aggregation was descriptive, focusing on frequency and proportional distribution across categories. Graphical representations were used to illustrate translational relationships between abstraction strategies, actuation types, and evidence levels. To enhance interpretability, extracted data were structured into four analytical layers representing the translational pathway from biological inspiration to clinical application (biological abstraction, engineering implementation, experimental validation, and technological maturity).

The four-layer analytical framework was selected because it captures the main translational stages through which biomimetic concepts evolve in orthosis development, from biological reference and abstraction to engineering realization, experimental validation, and technological maturity. Rather than adopting a generic evidence-synthesis taxonomy, this structure was designed to reflect the specific logic of biomimetic design processes and their progression toward clinical application.

Generative artificial intelligence tools were used exclusively for language refinement, graphic formatting, and creating a basis for the graphic summary. All methodological decisions, screening, data extraction, and analytical interpretation were conducted by the authors.

In addition to categorical synthesis, recurring patterns of methodological fragmentation identified across the four analytical layers were examined to gain higher-order structural insights. These insights informed the formulation of a structured biomimetic workflow that is presented in Section 4. The workflow was developed as an integrative conceptual outcome based on the gaps observed in abstraction traceability, engineering translation consistency, and validation maturity.

## 3. Results and Discussion

### 3.1. Study Selection 

A total of 453 records were identified through database searching. After duplicate removal, 378 records remained for screening. During title and abstract screening, records were classified into three predefined categories:

(i) Biomimetic orthoses, defined as orthotic devices explicitly developed using biological inspiration and abstraction;
(ii) Biomimetic materials, tools, or mechanisms relevant to orthosis development, referring to biologically inspired components or design strategies applicable to orthotic systems;
(iii) Non-eligible studies, comprising publications that were neither orthotic nor biomimetic.

Based on this screening stage, 29 records were assigned to category (i), 71 to category (ii), and 278 to category (iii). Full-text assessment was conducted for all records classified under categories (i) and (ii). Following eligibility verification, 10 studies met the criteria for biomimetic orthoses (category I), and four studies met the criteria for biomimetic materials or mechanisms relevant to orthotic development (category II). The remaining records were excluded after full-text evaluation.

**Figure 1:**
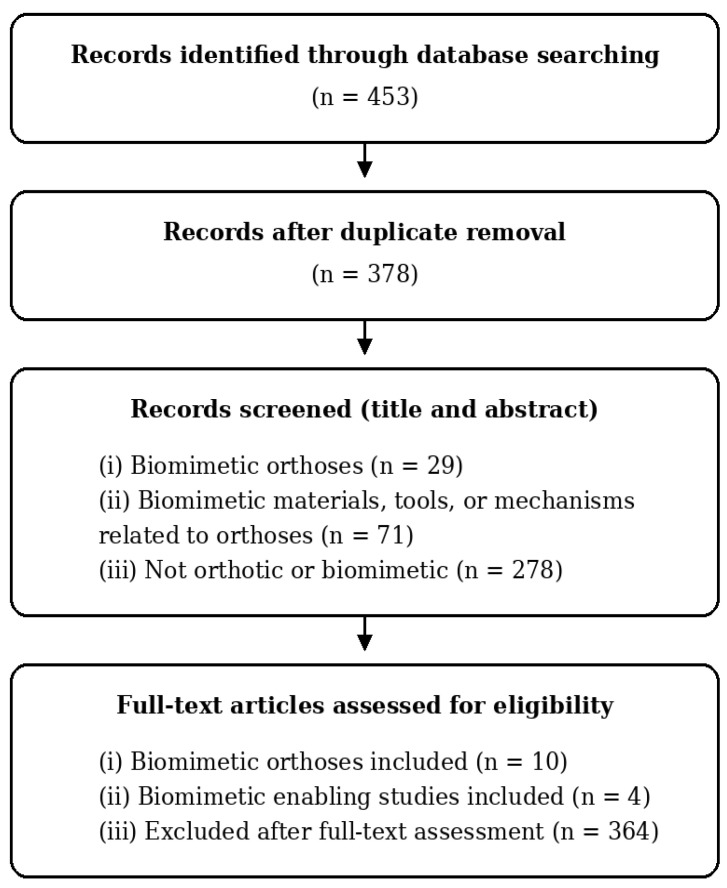
Flow diagram PRISMA (2020) [37] of the study selection process, illustrating record identification, screening, eligibility assessment, and final inclusion according to biomimetic relevance.

The final inclusion of 14 studies reflects both the novelty of the field and the strict requirement for explicit biomimetic terminology, suggesting that biomimetic orthosis research remains limited in volume and conceptual consolidation. This low yield also indicates that “biomimetic/bionic/bio-inspired” is frequently used as a broad descriptor, while only a small subset of publications provides sufficient methodological specificity to support extraction and cross-study synthesis. The complete selection process is illustrated in Figure 1. This terminology-based strategy may also have excluded orthotic systems that incorporate biologically inspired principles without explicitly describing the design process as biomimetic or bioinspired. Accordingly, the present review should be interpreted as a mapping of explicitly labelled biomimetic orthosis research rather than as an exhaustive representation of all orthotic systems that may implicitly draw on biological analogies.

### 3.2. Structure of Analytical Synthesis

Data extraction from studies classified under categories (i) and (ii) yielded the results summarized in Table 1, Table 2, Table 3 and Table 4. To enable a structured interpretation of biomimetic orthosis development, the extracted data were organized into four analytical layers reflecting the translational pathway from biological inspiration to technological and clinical application.

Table 1 (Biological Abstraction Framework) covers the foundational biomimetic layer, including biological reference models, levels of abstraction (morphological, functional, control-based, and material-inspired), anatomical targets, and the focus of abstraction. Table 2 (Engineering Implementation Strategies) addresses the translation of biological principles into technical systems, including orthosis classification, actuation category, energy source, structural architecture, and personalization strategy. Table 3 (Experimental Validation Characteristics) summarizes validation-related parameters, including validation level, participant characteristics, sample size, and the scope of follow-up. Table 4 (Functional Outcomes and Limitations) presents reported objectives, observed outcomes, technological maturity, and recurrent technical and clinical limitations.

This layered organization enables cross-comparison between abstraction strategy, engineering realization, and validation maturity, thereby exposing structural asymmetries within the translational pipeline. Importantly, the four-layer structure was adopted to preserve traceability from biological rationale to validation outcomes, since many studies report “bioinspiration” at a high level while under-specifying the intermediate steps that link biology to engineering implementation and experimental evidence.

### 3.3. Publication Activity over Time 

**Figure 2:**
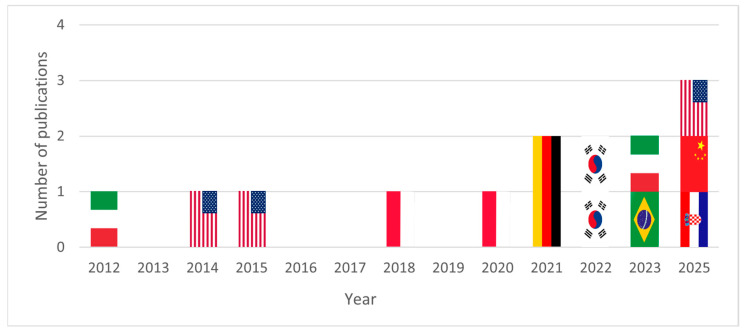
Annual distribution of included publications on biomimetic orthosis development. Flags indicate the countries of origin of the publications: United States, Brazil, South Korea, China, and Croatia.

The earliest publication meeting the inclusion criteria was identified in 2012. Between 2012 and 2020, publication activity was sporadic, with isolated studies emerging at irregular intervals. From 2021 onward, research activity increased modestly, with two studies published annually between 2021 and 2023. No eligible publications were identified in 2024, whereas three publications were recorded in 2025. Although the absolute number of studies remains limited (*n* = 14), the temporal distribution suggests a gradual increase in interest in biomimetic orthosis development over the past decade (Figure 2). However, because annual counts are low and sensitive to single-study fluctuations, this pattern should be interpreted as increased recent activity within the term-explicit subset captured by the search, rather than as a robust estimate of field-wide growth. The limited volume and uneven progression indicate that the development of biomimetic orthoses has not yet matured into a consolidated field of research, remaining mainly exploratory.

### 3.4. Biological Abstraction Patterns 

Data extraction revealed a clear predominance of human-based biological reference models as summarized in Table 1, Table 2, Table 3 and Table 4. One possible explanation for this predominance is that orthotic devices are designed to interface directly with human anatomy and movement patterns. Consequently, designers often prioritize human biomechanical analogies that closely replicate musculoskeletal functions, facilitating mechanical compatibility and improving the human–device interface. Eleven of the 14 included studies drew directly from human anatomical or neuromuscular systems, particularly muscle–tendon architectures, joint kinematics, and ligament behavior [39,40,41,42,43,46,47,48,49,50,52], while only 3 relied on non-human organisms, including insect wing joints, twining plant mechanisms, and ungulate ligament systems [44,45,51]. This pattern suggests that biomimetic abstraction in orthosis research remains strongly grounded in functional analogies with the human musculoskeletal system rather than broader cross-species biological strategies.

Human-based models were primarily associated with objectives focused on restoring impaired function [40,43,46,48,49] and preserving or enabling natural movement patterns [41,43,47,50,52]. Whereas non-human inspirations were aligned with mechanically advantageous behaviors (e.g., passive self-adjustment or stress absorption) that are less directly tied to human anatomical replication.

**Figure 3:**
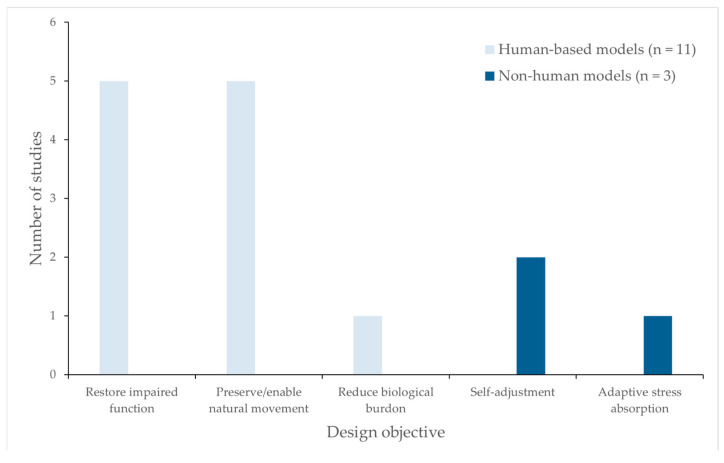
Divergence in design objectives between human-based and non-human biological inspirations in biomimetic orthosis research (*n* = 14 studies).

Notably, all non-human-inspired designs were implemented as passive systems, suggesting a conceptual separation between active biomechanical replication (human-based) and passive adaptive strategies (non-human-based). A smaller subset of studies addressed biological load reduction, emphasising load relief strategies rather than direct movement restoration [41] (Figure 3).

Across abstraction levels, functional translation of biomechanical principles predominated. Designs frequently reproduced tendon coordination, antagonistic muscle dynamics, rolling–sliding joint behavior, or ligament-like elastostatic responses. Control-based abstraction strategies, centered on neuromuscular activation patterns or movement synergies, were identified in only one study [39], while material-inspired approaches involving compliance or stimuli-responsive behavior were comparatively rare. In contrast, non-human-inspired designs were concentrated on objectives related to self-adjustment and adaptive stress absorption. Rather than replicating anatomical structures, these systems abstracted broader functional behaviors observed in biological organisms. This distribution suggests that the term “biomimetic” is predominantly operationalised as emulation of biomechanical functions (especially in human-inspired studies), while multiscale material intelligence and control-oriented biomimetics remain underrepresented.

Morphological abstraction alone was uncommon and generally appeared in combination with functional strategies. Taken together, the distribution of objectives and abstraction types indicates that biomimetic orthosis development remains strongly oriented toward human biomechanical analogues, with comparatively limited exploration of adaptive material intelligence or multi-scale biological strategies.

Additionally, studies inspired by non-human models tended to articulate the abstraction focus more explicitly (e.g., the targeted adaptive function), whereas several human-based studies used biomimetic terminology in a more general sense, with limited detail on how the biological principle was formalized into design requirements.

### 3.5. Engineering Translation Strategies 

Analysis of engineering implementation revealed that biological inspiration was most often translated into cable-driven electromechanical systems, particularly for devices inspired by muscles and tendons [39,40,41,43,46,48,49,52]. Cable-based electromechanical actuation appeared in five studies, making it the most dominant implementation strategy. Twisted-string actuators and pneumatic artificial muscles were less frequent but reflected attempts to replicate muscle-like force-length behaviour. Purely passive mechanical implementations were seen in structural or ligament-inspired designs. Stimulus-responsive 4D printed systems represented the most conceptually innovative category but remained largely at the conceptual or simulation stage.

Despite their popularity, cable-driven systems also present limitations, including friction losses, maintenance requirements, and limited adaptability to complex multi-joint dynamics. Emerging alternatives include soft robotic actuators, variable-stiffness structures, and material-based adaptive systems inspired by biological tissues.

**Figure 4:**
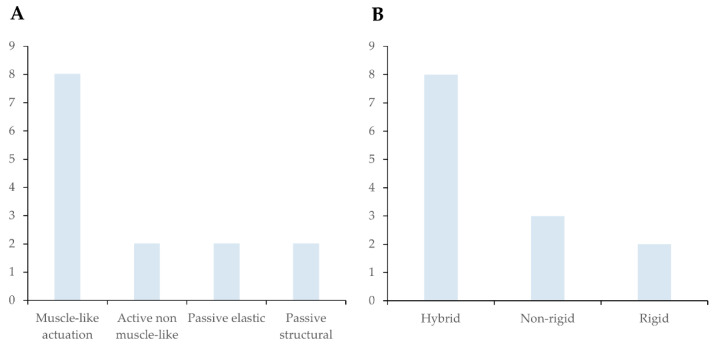
(**A**) Types of intervention in the included studies (*n* = 14). (**B**) Structural concepts in the included studies (*n* = 13), excluding study [39], because it was a control strategy with several possible forms.

In contrast, active approaches that do not rely on muscle-like analogues, such as rigid motor-driven systems without a traction contraction principle, have been reported in two studies [47,50]. Furthermore, two studies [44,45] employed entirely passive or stimulus-responsive passive structural solutions, emphasizing material or structural adaptation rather than active force generation. In addition, two studies report passive elastic mechanisms [42,51] (Figure 4A). Together, these findings indicate a strong preference for biologically inspired tensile actuation principles over alternative active or passive mechanical strategies. Overall, engineering translation has converged on a narrow set of actuation paradigms, primarily tensile and motor-driven systems, despite the broader conceptual diversity of biological inspirations reported in Table 1.

Hybrid architectures that combine flexible interfaces with rigid transmission components were predominant [40,42,43,45,46,48,49,50], while fully flexible architectures were reported in only three studies [41,51,52]. Fully rigid structures were identified mainly in passive splint concepts [44,47] (Figure 4B). This distribution suggests a convergence in engineering translation toward a limited set of performance paradigms, despite the conceptual diversity of biological inspiration. Such convergence may reflect practical constraints common to wearable orthoses, including portability, controllability, manufacturability, and user safety. However, it may also indicate limited exploration of alternative pathways for achieving bioinspired performance and material intelligence within the current evidence base.

Customization strategies were heterogeneous. Notably, the predominance of generalized devices indicated that many studies prioritized mechanical feasibility and proof-of-concept performance over workflow development for individualized clinical implementation. Most systems were classified as generalized or adjustable in size, while truly individualized designs, explicitly tailored through parametric modeling or custom fitting, were lacking. Overall, the translation from biology to engineering was technologically creative but lacked standardization and consistent reporting of abstraction.

### 3.6. Experimental Validation Characteristics

The maturity of validation across studies was limited. Most investigations involved prototype-level testing with small samples of participants. Among studies that reported sample size, the average was five participants, and several investigations relied exclusively on healthy volunteers rather than clinical populations. In many cases, evaluations were limited to feasibility testing or laboratory performance assessments rather than structured clinical evaluations. No studies reported longitudinal follow-up, randomized clinical trials, or multicenter validation. Overall, these findings indicate that research on biomimetic orthoses remains primarily focused on early-stage engineering validation, with limited progress toward clinical trials or real-world implementation. Taken together, these results suggest that most biomimetic orthosis concepts remain in early stages of translation, where engineering feasibility and functional principles are explored prior to more extensive clinical investigation.

With respect to personalization, eleven [39,40,41,42,43,44,45,49,50,52] studies described generalized systems intended for broad applicability. Three studies [46,47,49] described individual-specific construction. These findings indicate that the field remains largely confined to feasibility demonstration rather than rigorous clinical validation. This imbalance between innovative engineering concepts and limited validation maturity is consistent with the technological maturity classifications reported in Table 4.

### 3.7. Functional Outcomes and Technological Maturity 

The reported functional objectives of the studies included restoration of movement, reduction in metabolic cost, reduction in misalignment, load relief, and self-adjusting adaptability. Although several studies demonstrated feasibility under controlled conditions, technological maturity was predominantly categorized as conceptual, prototype, or early clinical trials. Only one study approached the level of a validated prototype under structured conditions [45]. Table 5 summarizes the methodological characteristics related to biomimetic development workflows. Notably, none of the studies included made explicit reference to an established biomimetic standard, such as ISO 18458 [11] or related frameworks. Overall, the completeness of the workflow across studies can therefore be characterized as predominantly partial, with only one study approaching substantial methodological completeness, namely the work by Cheng et al. (2021) [45], which presents an ISO-like biomimetic workflow structure, including stages of biological analysis, abstraction, and technical transfer. Five studies [44,48,49,50,51] explicitly described all three steps, although not in accordance with the standardized terminology or formal workflow structure. The remaining studies [39,40,41,42,43,45,46,47] provided only partial descriptions, with at least one step addressed implicitly or insufficiently detailed. In several cases, biological inspiration was stated, but the analytical path linking biological observation to technical implementation was not systematically articulated.

This pattern reveals a structural translational gap: while biological inspiration is frequently invoked, the methodological pathway linking biological observation to engineering realization is often insufficiently formalized. As a result, biomimetic contributions are difficult to compare across studies, and design rationales are not readily reusable or auditable as a methodological workflow. Overall, the workflow completeness across studies can therefore be characterized as predominantly partial, with only one study approaching substantial methodological completeness.

Experimental validation limitations, combined with restricted anatomical coverage (predominantly upper limb), further indicate that biomimetic orthosis development remains at an early stage of clinical translation. Most investigations were limited to single-session feasibility testing or short-term performance assessment. Among the studies reporting sample size, the average number of participants was five, with only one study [40] including more than ten participants (*n* = 14). Healthy individuals predominated as test populations, while exclusive patient-based validation was rare. This imbalance suggests that many devices remain at the prototype or preliminary clinical feasibility stage rather than advancing toward robust clinical validation.

Anatomical and functional coverage was similarly restricted. Upper-limb applications were predominant, particularly targeting hand, wrist, and forearm function. Lower-limb studies were fewer and primarily focused on the knee, while axial applications, such as cervical and lumbar regions, were minimally represented. No study identified trunk or head orthoses as primary application domains. The concentration on a limited set of anatomical targets, combined with small sample sizes and short-term testing, indicates that biomimetic orthosis research remains largely exploratory and has yet to achieve broad translational maturity.

Taken together, the results summarized in Table 5 and the corresponding validation characteristics suggest that the development of biomimetic orthoses is currently driven more by conceptual and mechanical innovation than by standardized methodological structuring or rigorous clinical evaluation. The absence of explicit reports on the biomimetic workflow and the predominance of small-scale feasibility tests highlight a critical opportunity for the field to adopt clearer methodological frameworks and more robust validation strategies to support clinical translation. To facilitate interpretation of the current state of the field, the distribution of studies across key stages of biomimetic development (biological analysis, abstraction, engineering implementation, experimental validation, and technological maturity) is compared and summarized in this section. The results indicate that progress in this literature is primarily driven by proof-of-concept or prototypes, rather than standardized biomimetic workflows associated with clinically robust evaluation pathways.

It should also be noted that prototype-level maturity is not unique to biomimetic orthosis research but is frequently observed across the broader field of wearable rehabilitation and emerging orthotic technologies. Accordingly, the immaturity identified here should be interpreted not only as a characteristic of biomimetic orthoses but also as part of a broader translational challenge in orthosis development.

### 3.8. Integrated Translational Analysis

**Figure 5:**
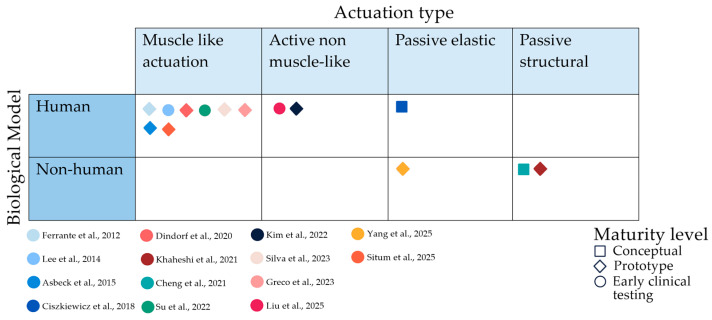
Translational Matrix Linking Biological Model, Engineering Implementation, and Evidence Maturity in Biomimetic Orthoses.

Figure 5 integrates the four analytical layers, mapping biological reference models in relation to engineering strategies and corresponding levels of experimental validation. The matrix reveals a striking concentration of muscle- and tendon-based abstractions that translate into cable-driven or electromechanical systems. These combinations were predominantly validated in prototypes or early stages of testing, often involving small samples and short-term evaluations. In contrast, non-human biological inspirations and material-based abstractions were represented less frequently and generally associated with lower validation maturity, including simulation-only studies or preliminary mechanical demonstrations. Notably, passive and material-inspired structural concepts rarely progressed beyond conceptual or feasibility validation. Given the limited number of studies included and the heterogeneity of design strategies, actuation mechanisms, and validation approaches, a formal statistical analysis of association was not considered methodologically robust. However, a frequency-based reading of the matrix suggests that functional abstractions derived from humans were more often associated with cable-driven or electromechanical implementations, while material- and non-human-inspired concepts were more commonly observed in conceptual, simulation-based, or mechanically validated stages.

This integrated analysis exposes a structural asymmetry within the translational pathway. While the spectrum of biological inspiration appears conceptually diverse, engineering implementation converges toward a limited set of actuation paradigms, primarily tensile and motor-driven systems. At the same time, validation maturity remains largely decoupled from abstraction complexity, with no study demonstrating progression to longitudinal or large-scale clinical validation.

Collectively, the matrix highlights a translational gap between the generation of biomimetic concepts and the development of clinically robust orthotic systems. The results suggest that innovation in this field is driven predominantly by mechanical feasibility rather than by a structured biomimetic methodology or systematic clinical consolidation. Importantly, the matrix also highlights that validation maturity does not systematically increase with abstraction sophistication; instead, conceptually different biomimetic inspirations often converge to similar levels of implementation and testing. This reinforces that, within the included evidence base, biomimetic diversity is more visible at the inspiration/abstraction level than at the implementation and clinical evidence levels.

## 4. Structured Biomimetic Workflow for Orthosis Development

**Figure 6:**
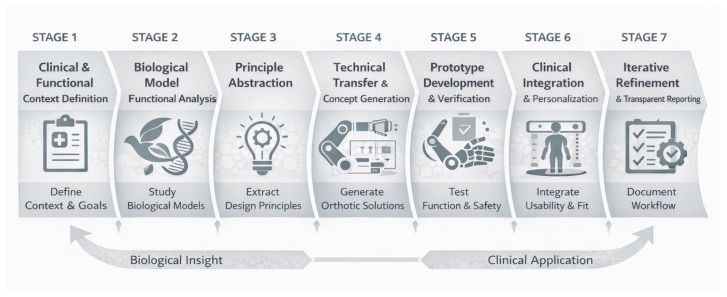
Conceptual framework of a structured biomimetic workflow for orthosis development.

The results of this review revealed substantial heterogeneity in how biomimetic principles are selected, abstracted, and transferred to orthotic engineering solutions. Although biological inspiration is often invoked, explicit methodological traceability between biological analysis and technical implementation is rarely documented. In response to these gaps, we propose a structured biomimetic workflow tailored to orthotic development, conceptually aligned with ISO 18458. The proposed framework organizes the development of biomimetic orthoses into seven sequential and traceable steps (Figure 6), ranging from clinical problem definition to iterative refinement.

- Stage 1—Clinical and Functional Context Definition: Orthosis development must begin with explicit definition of clinical indication, anatomical region, functional objective, user constraints, and personalization requirements. This stage establishes measurable performance criteria and system boundaries.
- Stage 2—Biological Model Identification and Functional Analysis: A biological system addressing an analogous functional challenge is selected. Emphasis is placed on functional mechanisms (force generation, compliance modulation, control strategy) rather than superficial morphological similarity.
- Stage 3—Principle Abstraction: Biological mechanisms are translated into abstract principles. It should be explicitly stated from which discipline the abstraction was obtained (morphological, functional, sensory, material, or multi-scale).
- Stage 4—Technical Transfer and Concept Generation: Abstract principles are transferred to orthotic design concepts, including performance category, structural architecture, power source, and customization strategy. Traceability between the biological reference and the engineering solution must be documented. For example, in the design of a biomimetic hand orthosis, the workflow begins with the analysis of human muscle and tendon mechanisms, followed by the abstraction of traction force transmission principles, engineering translation into cable-driven systems, and iterative validation through prototype testing.
- Stage 5—Prototype Development and Verification: Mechanical, functional, and safety validation are performed. Evaluation should assess whether the intended biological function has been successfully reproduced.
- Stage 6—Clinical Integration and Personalization: Fit, comfort, adherence, and scalability are evaluated. Digital workflows (e.g., 3D scanning and parametric modeling) should be incorporated where applicable.
- Stage 7—Iterative Refinement and Transparent Reporting: Documentation of biological analysis, abstraction logic, and validation outcomes ensures reproducibility and auditability.

This workflow does not prescribe a single design pathway but provides a structured methodological scaffold intended to enhance transparency, comparability, and clinical maturity in biomimetic orthosis research. By linking clinical problem definition, biological function analysis, abstraction level, technical transfer, and validation strategy, the framework addresses the methodological fragmentation identified in the reviewed literature. It enables clearer traceability between biological inspiration and engineering realization, reduces ambiguity in biomimetic claims, and supports reproducible reporting practices. Importantly, the workflow also facilitates alignment between biomimetic innovation and established orthotic requirements, including personalization, usability, safety, and scalability. In doing so, this contributes to bridging the gap between conceptual biomimetic inspiration and clinically robust orthotic implementation.

The proposed workflow should therefore be interpreted as a conceptually derived evaluative scaffold rather than as a validated benchmark. Future studies should apply it retrospectively to published cases and prospectively to ongoing orthosis development projects in order to test its practical discriminative value and methodological usefulness.

## 5. Conclusions

This systematic review mapped biomimetic strategies in orthosis development across four analytical layers: biological abstraction, engineering translation, experimental validation, and technological maturity. The findings indicate that biomimetic approaches are present in orthotic research but remain methodologically heterogeneous and clinically immature within the term-explicit literature captured by the search.

Human-based biological models, particularly musculotendinous systems, predominate, and abstraction is conducted mainly at the functional level. In contrast, non-human and material-inspired strategies are less frequent and usually associated with passive concepts. Despite the conceptual diversity in biological references, engineering implementation converges on a limited set of performance paradigms, most notably cable-driven and electromechanical systems. Experimental validation is largely feasibility-oriented, using small sample sizes, limited patient inclusion, lack of longitudinal follow-up, and restricted anatomical coverage.

An important structural gap identified is the lack of explicit and traceable biomimetic workflows. Biological analysis, abstraction, and technical transfer are often described implicitly, limiting methodological transparency and comparability. In response to this imbalance, a structured biomimetic workflow, conceptually aligned with ISO 18458, is presented to improve traceability, reporting clarity, and clinical progression. Future research should focus on the systematic integration of biomimetic methodologies into orthotic development, improving the reporting of biological abstraction processes, and advancing validation protocols for larger-scale clinical evaluations.

## Figures and Tables

**Table 1:** Biological Abstraction Framework.

Study	Title	Biological Model	Level of Abstraction	Anatomical Region	Abstraction Focus
Ferrante et al., 2012 [39]	Biomimetic NMES Controller for Arm Movements Supported by a Passive Exoskeleton *	Human motor control strategy	Control-based	Upper limb (shoulder and elbow)	Synergy-based muscle activation control
Lee et al., 2014 [40]	Development of a BiomHED for Restoration of Functional Hand Movement Post-Stroke *	Human muscle-tendon coordination patterns	Functional	Hand (fingers and thumb)	Multi-joint coordination and grasp synergy
Asbeck et al., 2015 [41]	A Biologically Inspired Soft Exosuit for Walking Assistance *	Human muscle-tendon-ligament system	Functional	Lower limb (ankle and hip)	Force transmission and energy storage during gait
Ciszkiewicz and Milewski 2018 [42]	Ligament-Based Spine-Segment Mechanisms **	Human lumbar ligament system (L4–L5)	Functional	Lumbar spine	Elastostatic ligament behavior modeling
Dindorf and Wos 2020 [43]	Using Bioelectric Signals to Control a Wearable Orthosis of the Elbow Joint with a Bimuscular Pneumatic Servo-Drive *	Human antagonistic muscle pair (biceps-triceps)	Control-based	Elbow	Neuromuscular activation strategy
Khaheshi et al., 2021 [44]	Spiky-Joint: A Bioinspired Solution to Combine Mobility and Support *	Insect wing vein joint	Material-inspired + morphological	Wrist	Load-dependent stiffness transition
Cheng et al., 2021 [45]	Bio-Inspired Motion Mechanisms: Computational Design and Material Programming of Self-Adjusting 4D-Printed Wearable Systems **	Twining plant (Dioscorea bulbifera)	Material-inspired	Forearm and wrist (example)	Hygroscopic shape-change force generation
Se et al., 2022 [46]	A Soft, Wearable Skin-Brace for Assisting Forearm Pronation and Supination with a Low-Profile Design *	Human forearm musculoskeletal system	Functional	Forearm and wrist	Muscle-tendon biomechanics for rotational degrees of freedom
Kim et al., 2022 [47]	Bioinspired Knee Joint of a Lower-Limb Exoskeleton for Misalignment Reduction *	Human knee anatomy (ligaments, meniscus)	Functional + morphological	Knee	Rolling-sliding kinematics and variable center of rotation
Silva et al., 2023 [48]	Biomimetic Design of a Tendon-Driven Myoelectric Soft Hand Exoskeleton for Upper-Limb Rehabilitation *	Human hand tendon-muscle system	Functional	Hand and wrist	Agonist-antagonist tendon coordination
Greco et al., 2023 [49]	Lightweight Bioinspired Exoskeleton for Wrist Rehabilitation Powered by Twisted and Coiled Artificial Muscles *	Human wrist muscle arrangement	Functional	Wrist	Flexor-extensor and deviation mechanics
Liu and He 2025 [50]	Design of a Biomimetic Knee Joint Orthosis for Motion Rehabilitation Assistance *	Human knee joint kinematics	Functional	Knee	Anatomical motion reproduction and misalignment reduction
Yang et al., 2025 [51]	Passive Neck Brace for Surgeons *	Ungulate nuchal ligament	Functional	Cervical spine	Passive load support based on elastic ligament principles
Situm et al., 2025 [52]	Mechatronic and Robotic Systems Utilizing Pneumatic Artificial Muscles as Actuators **	Human skeletal muscle	Functional	Multiple regions	Muscle-like contractile force generation

* Category (i): Biomimetic orthoses, defined as orthotic devices explicitly developed through biological inspiration and abstraction. ** Category (ii): Biomimetic materials, tools, or mechanisms relevant to orthosis development, referring to biologically inspired components or design strategies applicable to orthotic systems.

**Table 2:** Engineering Implementation Strategies.

Study	Orthosis Classification	Actuation Mechanism	Energy Source	Structural Architecture	Personalization Strategy
[39]	Passive upper-limb exoskeleton with controller	NMES-based control	Electrical stimulation	Rigid passive exoskeleton	Generalized
[40]	Hand exotendon rehabilitation device	Cable-driven electromechanical	Electric motors	Hybrid (soft glove and rigid forearm unit)	Generalized (subject-specific tuning)
[41]	Soft wearable orthosis (exosuit)	Cable-driven electromechanical	Electric motors	Soft textile-based architecture with external transmission	Generalized (adjustable fit)
[42]	Spine-segment mechanical module	Purely passive mechanical	Mechanical springs and cables	Hybrid (rigid platforms and flexible elements)	Generalized (model-based)
[43]	Active elbow orthosis	Pneumatic artificial muscle	Compressed air	Hybrid (rigid frame and soft pneumatic muscles)	Generalized
[44]	Wrist splint with controlled mobility	Purely passive mechanical	None (mechanical interlocking)	Rigid 3D-printed polymer structure	Generalized
[45]	Self-adjusting wearable orthosis concept	4D stimuli-responsive	Environmental stimuli (humidity)	Hybrid layered polymer composite	Generalized (adaptable design strategy)
[46]	Wearable forearm rehabilitation orthosis	Cable-driven electromechanical	Remote electric motor	Hybrid (soft interface and semi-rigid frame)	Individual-specific (parameter-based 3D print)
[47]	Lower-limb knee exoskeleton	Cable-driven electromechanical	Electric actuator	Rigid mechanical joint mechanism	Individual-specific (custom-fitted frame)
[48]	Soft hand exoskeleton	Twisted-string actuator	Electric motor	Hybrid (soft glove and semi-rigid base)	Generalized (size-adjustable)
[49]	Wrist rehabilitation exoskeleton	Twisted-string actuator	Electrothermal	Hybrid (soft artificial muscles and semi-rigid supports)	Individual-specific (scalable parameters)
[50]	Active knee orthosis/exoskeleton	Cable-driven electromechanical	Electric motor	Hybrid (rigid cam-based joint and flexible transmission)	Generalized
[51]	Passive cervical orthosis	Elastic passive	Elastic mechanical energy	Soft elastic structure	Generalized
[52]	Orthosis-relevant actuation systems	Pneumatic artificial muscle	Compressed air	Soft pneumatic muscle structures	Generalized

**Table 3:** Experimental Validation Characteristics.

Study	Validation Level	Sample Size	Population Type	Clinical Condition	Follow-Up Duration	Validation Scope
[39]	Healthy volunteer testing	2	Healthy participants	None	Not reported	Tracking performance
[40]	Mixed human testing	14	Patients and healthy participants	Post-stroke	Not reported	Reachable workspace and pinch kinematics
[41]	Healthy volunteer testing	5	Healthy participants	None	Not reported	Energetic and kinematic performance
[42]	Healthy volunteer testing	2	Healthy participants	None	Not reported	Tracking performance
[43]	Healthy volunteer testing	Not reported	Healthy participants	None	Not reported	Motion control performance
[44]	Prototype mechanical testing	Not reported	No human participants	None	Not applicable	Mechanical load-transition behavior
[45]	Simulation only	Simulation only	None	None	Not applicable	Computational elastostatic modeling
[46]	Mixed human testing	9	Patients and healthy participants	Neurological impairment (stroke)	Not reported	Range of motion and functional performance
[47]	Single-subject testing	1	Not reported	Not specified	Not reported	Kinematic alignment
[48]	Healthy volunteer testing	5	Healthy participants	None	Not reported	Functional grasp and EMG control
[49]	Healthy volunteer testing	3	Healthy participants	Occupational strain	Not reported	Muscle effort reduction
[50]	Patient testing	4	Patients	Rehabilitation context	Not reported	Kinematic consistency
[51]	Healthy volunteer testing	3	Healthy participants	Occupational strain	Not reported	Muscle effort reduction
[52]	Limited prototype testing in humans	Not reported	Healthy participants	None	Not reported	Feasibility demonstration

**Table 4:** Functional Outcomes and Limitations.

Study	Primary Functional Objective	Demonstrated Outcome	Maturity Level	Main Technical Limitation	Main Clinical Limitation
[39]	Support upper-limb movements through biomimetic control	Feasibility of movement tracking was demonstrated	Prototype	Limited adaptability; sensitivity to fatigue	Validated mainly in healthy participants
[40]	Restore functional hand movement after stroke	Improved reachable workspace and pinch kinematics	Early clinical testing	Limited independent finger control	Pilot-scale evidence only
[41]	Reduce metabolic cost during walking	Reduced energy expenditure and preserved gait kinematics were demonstrated in controlled trials	Validated prototype	Bulkiness, transmission hysteresis, and non-portable setup	No patient testing and no longitudinal outcomes
[42]	Reproduce lumbar elastostatic behavior	Target behavior was reproduced computationally	Conceptual	Numerical validation only; no full prototype	No human testing
[43]	Enable bioelectric-controlled elbow assistance	Controlled elbow motion was demonstrated under laboratory conditions	Prototype	Sensitivity to signal noise; pneumatic nonlinearities	No patient validation
[44]	Combine mobility and support	Load-dependent stiffness transition was validated mechanically	Prototype	Limited scalability in degrees of freedom; material durability	No human testing
[45]	Enable passive self-adjusting orthoses	Programmable shape-change behavior was demonstrated	Conceptual	Environmental sensitivity; limited force output	No validation within a clinical workflow
[46]	Assist forearm rotation during rehabilitation	Improved range of motion was observed in a small user trial	Early clinical testing	Remote actuation; limited portability	Small cohort and short-term testing only
[47]	Reduce joint misalignment	Reduced misalignment indicators were observed in controlled evaluation	Prototype	Simplified kinematic modeling; limited three-dimensional validation	No robust patient validation
[48]	Restore grasp function	Functional grasp assistance was demonstrated under controlled conditions	Prototype	Limited force output; underactuated fingers	Tested mainly in healthy participants
[49]	Provide lightweight wrist assistance	Feasibility of wrist motion assistance was demonstrated	Prototype	Thermal constraints; actuator durability	No clinical outcome validation
[50]	Reproduce anatomical knee motion	Improved kinematic consistency was observed in small trials	Early clinical testing	Simplified motion assumptions	Limited patient sample and no long-term data
[51]	Reduce neck muscle load during sustained flexion	Short-term reduction in muscle effort was demonstrated	Prototype	Fit stability; ergonomic integration	No long-term occupational validation
[52]	Demonstrate the feasibility of muscle-like actuation	Feasibility was demonstrated in robotic and assistive contexts	Prototype	Control complexity; air supply requirements	Not clinically validated

**Table 5:** Methodological Transparency of Biomimetic Reporting in Included Studies (*n* = 14).

Study	Reference to Biomimetics Standard ISO 18458	Biological Function Analysis	Abstraction Process Description	Technical Transfer Description	Overall Workflow Completeness
Ferrante et al., 2012 [39]	Not referenced	Partially described	Partially described	Partially described	Partial
Lee et al., 2014 [40]	Not referenced	Partially described	Partially described	Explicitly described	Partial
Asbeck et al., 2015 [41]	Not referenced	Partially described	Partially described	Explicitly described	Partial
Ciszkiewicz and Milewski, 2018 [42]	Not referenced	Partially described	Partially described	Partially described	Partial
Dindorf and Wos, 2020 [43]	Not referenced	Explicitly described	Partially described	Explicitly described	Partial
Khaheshi et al., 2021 [44]	Not referenced	Explicitly described	Explicitly described	Explicitly described	Partial (no standardized structure)
Cheng et al., 2021 [45]	ISO-like structure (not formally cited)	Partially described	Partially described	Partially described	Substantially complete
Se et al., 2022 [46]	Not referenced	Partially described	Partially described	Partially described	Partial
Kim et al., 2022 [47]	Not referenced	Partially described	Partially described	Partially described	Partial
Silva et al., 2023 [48]	Not referenced	Explicitly described	Explicitly described	Explicitly described	Partial (no standardized structure)
Greco et al., 2023 [49]	Not referenced	Explicitly described	Explicitly described	Explicitly described	Partial (no standardized structure)
Liu and He, 2025 [50]	Not referenced	Explicitly described	Explicitly described	Explicitly described	Partial (no standardized structure)
Yang et al., 2025 [51]	Not referenced	Explicitly described	Explicitly described	Explicitly described	Partial (no standardized structure)
Situm et al., 2025 [52]	Not referenced	Partially described	Partially described	Partially described	Partial

## Data Availability

The data presented in this study are available upon request from the corresponding authors. The data are not publicly available for confidentiality reasons.

## Supplementary Materials

The following supporting information can be downloaded at https://www.mdpi.com/article/10.3390/biomimetics11040241/s1. Table S1: Data Extraction Template.

## Abbreviations

The following abbreviations are used in this manuscript:

BioMHED	Biomimetic Hand
DOF	Degrees of freedom
NMES	Neuromuscular Electrical Stimulation
PICOC	Population, Intervention, Comparison, Outcome, Context
PRISMA	Preferred Reporting Items for Systematic Reviews and Meta-Analyses

## References

[1] Mohaddis M., Maqsood S.A., Ago E., Singh S., Naim Z., Prasad S. (2023). Enhancing functional rehabilitation through orthotic interventions for foot and ankle conditions: A narrative review. Cureus.

[2] (2020). Prosthetics and Orthotics—Vocabulary—Part 1: General Terms for External Limb Prostheses and External Orthoses.

[3] World Health Organization (2024). Assistive Technology. https://www.who.int/news-room/fact-sheets/detail/assistive-technology.

[4] Dabnichki P., Pang T.Y. (2025). User-centered design framework for personalized ankle–foot orthoses. Prosthesis.

[5] Swinnen E., Kerckhofs E. (2015). Compliance of patients wearing an orthotic device or orthopedic shoes: A systematic review. J. Bodyw. Mov. Ther..

[6] Bashir A.Z., Dinkel D.M., Pipinos I.I., Johanning J.M., Myers S.A. (2022). Patient compliance with wearing lower limb assistive devices: A scoping review. J. Manip. Physiol. Ther..

[7] Resch S., Schauer J., Schwind V., Völz D., Sanchez-Morillo D. (2025). Improving social acceptance of orthopedic foot orthoses through image-generative AI in product design. Appl. Sci..

[8] Devanand D.B., Gardiner M.D., Kedgley A.E. (2025). A compact orthosis compliance monitoring device using pressure sensors and accelerometers: Design and proof-of-concept testing. Sensors.

[9] Wang J.Z., Wojciechowski E.A., Paine T., Burns J., Cheng T.L. (2025). Feasibility of designing, manufacturing and delivering 3D printed ankle-foot orthoses: An updated systematic review. J. Foot Ankle Res..

[10] Alrasheedi N.H., Tlija M., Elloumi N., Louhichi B. (2024). A critical review of 3D printed orthoses towards workflow implementation in clinical practice. J. Eng. Res..

[11] (2015). Biomimetics—Terminology, Concepts and Methodology.

[12] (2016). Biomimetics—Biomimetic Materials, Structures and Components.

[13] (2015). Biomimetics—Biomimetic Structural Optimization.

[14] Bar-Cohen Y. (2005). Biomimetics: Biologically Inspired Technologies.

[15] Vincent J.F.V., Bogatyreva O.A., Bogatyrev N.R., Bowyer A., Pahl A.-K. (2006). Biomimetics: Its practice and theory. J. R. Soc. Interface.

[16] Bhushan B. (2009). Biomimetics: Lessons from nature—An overview. Philos. Trans. R. Soc. A Math. Phys. Eng. Sci..

[17] Sauer A., Beismann H., Jäger M., Hamm C. (2018). Bionik in der Strukturoptimierung: Praxishandbuch für Ressourceneffizienten Leichtbau.

[18] Arena P., Bucolo M., Buscarino A., Fortuna L., Frasca M. (2021). Reviewing bioinspired technologies for future trends: A complex systems point of view. Front. Phys..

[19] Fattepur G., Patil A.Y., Kumar P., Kumar A., Hegde C., Siddhalingeshwar I.G., Kumar R., Khan T.M.Y. (2024). Bio-inspired designs: Leveraging biological brilliance in mechanical engineering—An overview. 3 Biotech.

[20] Varaganti P., Seo S. (2024). Recent advances in biomimetics for the development of bio-inspired prosthetic limbs. Biomimetics.

[21] Tanczak N., Yurkewich A., Missiroli F., Wee S.K., Kager S., Choi H., Cho K.-J., Yap H.K., Piazza C., Masia L. (2025). Soft Robotics in upper limb Neurorehabilitation and Assistance: Current Clinical Evidence and Recommendations. Soft Robot..

[22] Almeida J.F., Santos C.P. (2025). Bio-inspired control strategies in wearable robotics: A comprehensive review of CPGs and DMPs. Annu. Rev. Control.

[23] Wang X., Wei R., Chen Z., Pang H., Li H., Yang Y., Hua Q., Shen G. (2025). Bioinspired intelligent soft robotics: From multidisciplinary integration to next-generation intelligence. Adv. Sci..

[24] Wang Z., Xiao C., Roy M., Yuan Z., Zhao L., Liu Y., Guo X., Lu P. (2023). Bioinspired skin towards next-generation rehabilitation medicine. Front. Bioeng. Biotechnol..

[25] Sharma B., Phan P.T., Davies J., Hoang T.T., Nguyen C.C., Ji A., Zhu K., Nicotra E., Lovell N.H., Do T.N. (2024). Soft upper-limb wearable robotic devices: Technology and applications. Adv. Intell. Syst..

[26] Pitta Kruize C., Panahkhahi S., Putra N.E., Diaz-Payno P., van Osch G., Zadpoor A.A., Mirzaali M.J. (2021). Biomimetic approaches for the design and fabrication of bone-to-soft tissue interfaces. ACS Biomater. Sci. Eng..

[27] Siddique S.H., Hazell P.J., Wang H., Escobedo J.P., Ameri A.A.H. (2022). Lessons from nature: 3D printed bio-inspired porous structures for impact energy absorption—A review. Addit. Manuf..

[28] Savolainen H., Hosseiniyan N., Piedrahita-Bello M., Ikkala O. (2025). Bioinspired nondissipative mechanical energy storage and release in hydrogels via hierarchical sequentially swollen stretched chains. Nat. Commun..

[29] Kunkel M.E., Sauer A., Isaacs C., Ganga T.A.F., Fazan L.H., Rorato E.K. (2025). Teaching bioinspired design for assistive technologies using additive manufacturing: A collaborative experience. Biomimetics.

[30] Du Plessis A., Broeckhoven C., Yadroitsava I., Yadroitsev I., Hands C.H., Kunju R., Bhate D. (2019). Beautiful and functional: A review of biomimetic design in additive manufacturing. Addit. Manuf..

[31] Kunkel M.E., Araújo A.C.C.P.S., Lombelo C.B., da Ana P.A. (2023). Narrative review on the application of additive manufacturing in the production of upper limb orthoses. Current Trends in Biomedical Engineering.

[32] Arafa M.A., Goher K. (2025). Toward bioinspired prostheses based on human hand anthropomorphic depiction: A review article. J. Robot..

[33] Guo K., Lu J., Wu Y., Hu X., Yang H. (2024). The latest research progress on bionic artificial hands: A systematic review. Micromachines.

[34] Lingampally P.K., Ramanathan K.C., Shanmugam R., Cepova L., Salunkhe S. (2024). Wearable assistive rehabilitation robotic devices—A comprehensive review. Machines.

[35] Wanieck K., Beismann H. (2021). Perception and role of standards in the world of biomimetics. Bioinspir. Biomim. Nanobiomater..

[36] Kitchenham B., Charters S. (2007). Guidelines for Performing Systematic Literature Reviews in Software Engineering. Keele University and Durham University Joint Report, Technical Report EBSE-2007-01. https://www.elsevier.com/__data/promis_misc/525444systematicreviewsguide.pdf.

[37] PRISMA Group Preferred Reporting Items for Systematic Reviews and Meta-Analyses. https://www.prisma-statement.org.

[38] Petticrew M., Roberts H. (2006). Systematic Reviews in the Social Sciences: A Practical Guide.

[39] Ferrante S., Ambrosini E., Ferrigno G., Pedrocchi A. (2012). Biomimetic NMES controller for arm movements supported by a passive exoskeleton. Proc. Annu. Int. Conf. IEEE Eng. Med. Biol. Soc..

[40] Lee S., Landers K., Park H. (2014). Development of a biomimetic hand exotendon device (BiomHED) for restoration of functional hand movement post-stroke. IEEE Trans. Neural Syst. Rehabil. Eng..

[41] Asbeck A., De Rossi S., Holt K., Walsh C. (2015). A biologically inspired soft exosuit for walking assistance. Int. J. Robot. Res..

[42] Ciszkiewicz A., Milewski G. (2018). Ligament-based spine-segment mechanisms. Bull. Pol. Acad. Sci. Tech. Sci..

[43] Dindorf R., Wos P. (2020). Using bioelectric signals to control a wearable orthosis of the elbow joint with bi-muscular pneumatic servo-drive. Robotica.

[44] Khaheshi A., Gorb S., Rajabi H. (2021). Spiky-joint: A bioinspired solution to combine mobility and support. Appl. Phys. A.

[45] Cheng T., Thielen M., Poppinga S., Tahouni Y., Wood D., Steinberg T., Menges A., Speck T. (2021). Bio-inspired motion mechanisms: Computational design and material programming of self-adjusting 4D-printed wearable systems. Adv. Sci..

[46] Su H., Lee K., Kim Y., Park H. (2022). A soft, wearable skin-brace for assisting forearm pronation and supination with a low-profile design. IEEE Robot. Autom. Lett..

[47] Kim T., Jeong M., Kong K. (2022). Bioinspired knee joint of a lower-limb exoskeleton for misalignment reduction. IEEE/ASME Trans. Mechatron..

[48] Silva R., Lourenco B.G., Ulhoa P.H., Dias E.A., da Cunha F.L., Tonetto C.P., Villani L.G., Vimieiro C.B., Lepski G.A., Monjardim M. (2023). Biomimetic design of a tendon-driven myoelectric soft hand exoskeleton for upper-limb rehabilitation. Biomimetics.

[49] Greco C., Weerakkody T., Cichella V., Pagnotta L., Lamuta C. (2023). Lightweight bioinspired exoskeleton for wrist rehabilitation powered by twisted and coiled artificial muscles. Robotics.

[50] Liu K., He J. (2025). Design of a biomimetic knee joint orthosis for motion rehabilitation assistance. IEEE/ASME Trans. Mechatron..

[51] Yang Z., Sathe T., Shah M., Shah J., Hu D. (2025). Passive neck brace for surgeons. Ann. N. Y. Acad. Sci..

[52] Situm Z., Benic J., Cipek M. (2025). Mechatronic and robotic systems utilizing pneumatic artificial muscles as actuators. Inventions.

