# Supplementary material for: Biomimetic Strategies in Orthosis Design: A Scoping Review of Biological Abstraction and Functional Translation
Source: Biomimetics (Basel). 2026 Apr 3;11(4):241. doi: 10.3390/biomimetics11040241 (PMC13113914; doi:10.3390/biomimetics11040241)
Supplement: Supplementary file 1 [file biomimetics-11-00241-s001.zip › biomimetics-4194340-supplementary.pdf]

Supplementary Table S1 – Data Extraction Template

Study	Biological Inspiration	Biological System	Abstraction Level	Engineering Translation	Actuation Principle	Structural Strategy	Experimental Validation	Technological Readiness (TRL)
Author, Year	Human muscle–tendon	Musculoskeletal system	Functional abstraction	Cable-driven mechanism	Cable-driven	Soft / hybrid structure	Prototype testing	TRL 3
